# Financial implications of treating nonsevere gram-negative clinical mastitis in 3 California dairies

**DOI:** 10.3168/jdsc.2024-0548

**Published:** 2024-06-28

**Authors:** D.R. Bruno, R.M. Cleale, M.W. Overton, T. Short, J.R. Pedraza, R. Wallace

**Affiliations:** 1 University of California Agriculture and Natural Resources, Cooperative Extension, Fresno, CA 93710; 2 Zoetis, Parsippany, NJ 07054

## Abstract

On 3 large California dairies, 415 lactating cows with nonsevere clinical mastitis (CM) and infected with gram-negative (GN) bacteria were randomly assigned to 1 of 3 treatment groups: nontreated control (CON; 135 cases), 2 d of ceftiofur HCl (SP2; 133 cases), or 5 d of ceftiofur HCl (SP5; 147 cases). Bacteriological cure, clinical cure, mastitis recurrence, culling or death, and overall treatment success differed among treatment groups. Although duration of milk withheld due to mastitis therapy was higher for SP5 (9.4 d), there was no difference between CON (6.9 d) and SP2 (7.1 d). Culling and death rates due to GN CM were the main effects that affected partial cost calculations. Of study cows culled across the 3 herds, a higher proportion of CON cows (25%) were culled compared with SP2 (11%) or SP5 (18%). Mastitis-related expenses were higher ($550) for CON than SP2 ($343) or SP5 ($423). Results of this partial budget evaluation for the 3 California dairies indicated economic justification for treating cases of nonsevere GN CM with ceftiofur HCl for 2 d.

## Highlights

• Mastitis-related expenses were higher for the controls than CM cases treated for 2 or 5 days.
• Treatment for 2 days with ceftiofur HCl reduced expenses by $207/CM case.
• Treatment for 5 days with ceftiofur HCl reduced expenses by $127/CM case.
• Treatment for 2 or 5 days yielded the same clinical and bacteriological responses.
• Treating nonsevere GN CM for 2 days was economically justified in the herds studied.

## Graphical Abstract

**Figure:**
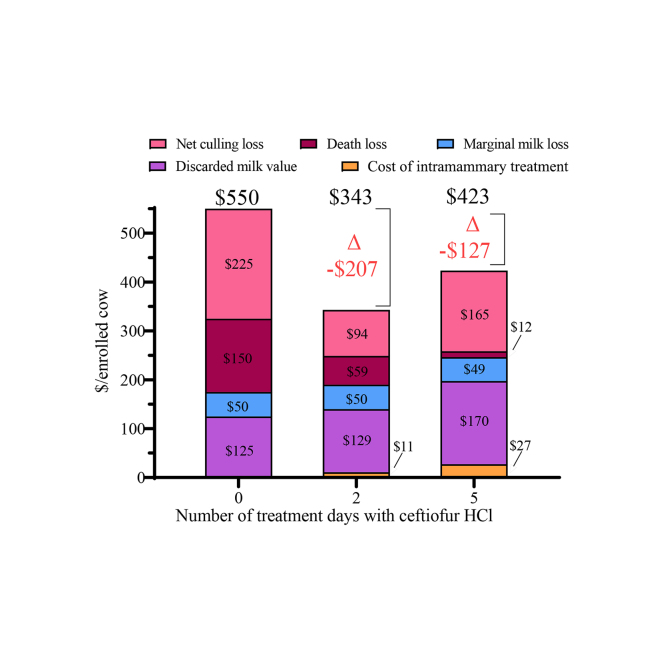
**Summary:** Bovine mastitis is an economically important disease that causes financial losses for dairy producers. A study was conducted to evaluate the biological impact of treating nonsevere gram-negative (GN) clinical mastitis (CM) with ceftiofur HCl for 2 or 5 days compared with nontreatment at 3 California dairies. This report used the outcomes from the study to perform a partial budget calculation of the economic impact of treating nonsevere GN CM. The partial budget evaluation showed mastitis-related expenses were higher for the nontreated group ($550) compared with animals treated with ceftiofur HCl, and were lower for the 2- ($343) compared with the 5-day ($423) treatment. The greatest differences between treatments were related to higher costs associated with the culling- and death-related expenses for nontreated cows compared with the treated groups that were incurred over the 90 days following GN mastitis diagnosis.

Bovine mastitis is a common and costly disease that negatively affects dairy farms (Cha et al., 2011) and can affect a cow's ability to remain productive and increase risk of early culling (Gröhn et al., 2004; Hertl et al., 2011). In the United States, mastitis-related economic losses are estimated at US$2 billion annually (The Cattle Site, 2020), with indirect costs accounting for >70% of the total costs (Rollin et al., 2015). Clinical mastitis (**CM**) affects 25% of lactating cows per dairy farm annually (USDA, 2018), and cases are classified according to their clinical presentation (Bruno et al., 2024). Lactating cows presenting with nonsevere (mild and moderate) mastitis are typically treated with antibiotics, whereas severe cases receive additional supportive therapy. Extensive use of antibiotics for mastitis therapy and associated concerns about antibiotic usage in food animals and potential risk of antibiotic resistance have prompted studies on the use of pathogen-specific mastitis therapy to promote judicious use of medically important antimicrobials (de Jong et al., 2023; Bruno et al., 2024). The goal of CM therapy is to alleviate illness and suffering of cows, restoring the affected quarter and milk to clinical normality while minimizing mastitis-related losses. The decision to treat CM must be cost effective, and the cost of discarded milk and medication must be less than the cost of nontreatment (Van Eenennaam et al., 1995). Limited information is available on the financial implications of mastitis therapy (Van Eenennaam et al., 1995; Cha et al., 2011; Rollin et al., 2015; Leite de Campos et al., 2023), and to our knowledge, no analysis has been presented specific to nonsevere gram-negative (**GN**) CM. This article presents a partial budget economic analysis of results from a study that reported responses following treatment of nonsevere CM caused by GN bacteria. The analysis is based on previously published data that demonstrated efficacy of 2 ceftiofur HCl therapy regimens (2 and 5 d) compared with nontreatment in dairy cattle from 3 California dairies (Bruno et al., 2024).

Study design and experimental methods were previously described (Bruno et al., 2024). Sample size and power calculations were based on data from previous studies (Schukken et al., 2011; Fuenzalida and Ruegg, 2019) and assumed one-sided significance at α = 0.05 and 80% power. Assuming a 26% unit difference in cure rate between ceftiofur and control groups, estimated sample size was 41 cases per treatment group per site and at least 3 sites. In brief, 415 cows exhibiting signs of mild or moderate CM, and confirmed to be infected with GN organisms by MALDI-TOF, were randomly assigned to 1 of 3 treatments: **SP2**: treatment with ceftiofur HCl (125 mg in 10 mL, Spectramast LC, Zoetis, Parsippany, NJ) at study enrollment and 24 h later for a total of 2 d; **SP5**: 5 treatments with ceftiofur HCl (125 mg in 10 mL, Spectramast LC, Zoetis, Parsippany, NJ) beginning on the day of enrollment and for the next 4 d at 24-h intervals; or **CON**: no treatment. Similarly to previous studies (Lago et al., 2011; Vasquez et al., 2016; Fuenzalida and Ruegg, 2019), study enrollment was based on on-farm culture (**OFC**) results, and outcomes with “time to event” were calculated based on enrollment day. Randomization (Kang et al., 2008) resulted in similar distribution of treatments across herds, and the description of GN CM cases by farm, quarter, severity, season, parity, and breed are summarized in Bruno et al. (2024). Aseptic milk samples were collected from CM cases 14, 21, and 28 ± 3 d after enrollment for bacteriological outcomes and milk components. Cows from all treatments were housed in each dairy's hospital. A milk withdrawal period of 72 h and a preslaughter withdrawal period of 48 h were followed after the last SP2 and SP5 treatment. Cows assigned to CON remained in the hospital until considered clinically well and without signs of udder inflammation or abnormal milk. Farm personnel or the research team, or both, observed cows until culling/death, dry off, or 90 d after enrollment, whichever came first.

Based on results of Bruno et al. (2024), derivative calculations were performed to estimate the partial budget value of implementing ceftiofur HCl treatment in cows with nonsevere GN CM. Inputs and assumptions for computations are defined as follows. Cost of product for GN CM treatment ($5.47/tube intramammary [**IMM**] ceftiofur HCl) was obtained from a retailer website (www.valleyvet.com). Cost of discarded milk from the time of CM diagnosis until milk could enter the bulk tank was defined for each cow as the mean elapsed time for clinical signs to resolve or 72 h after the last antibiotic tube was administered, whichever was longer, multiplied by average daily milk yield. At the time of enrollment, mean milk yield across treatments was 41.1 kg/d, and mean days of milk withholding were 6.9, 7.1, and 9.4 d for CON, SP2, and SP5, respectively. Only cows with a DHIA test day result before the day of enrollment were included in computation of pretreatment milk yield. Following expiration of the milk withholding period, milk production losses per cow due to GN CM through 90 d post-treatment were computed for individual cows; over 90 d following enrollment, mean milk production averaged 39.3 kg/d. It was assumed that milk yield was reduced 5% due to GN CM over the period from expiration of the milk withholding period through 90 d, or until the time a cow died or was culled. Estimated reduction in milk yield over a 70-d window following GN CM caused by *Escherichia coli* or *Klebsiella* spp. documented by Gröhn et al. (2004) was at least 10.8%. Schukken et al. (2009) showed that cases of nonsevere GN CM reduced milk yield by at least 12% over 70 d following diagnosis. More recently, Heikkilä et al. (2018) showed that *E. coli* mastitis diagnosed before peak lactation caused a 10.6% reduction in 305-d milk yield. Based on the citations, our estimate of a 5% reduction in milk yield over a 90-d assessment period is conservative.

A historical milk value of $20/45.4 kg ($0.44/kg) was assumed throughout calculations. To reflect that future milk reductions occurred in the absence of incremental feed input cost, the value of milk loss was discounted to account for the absence of associated feed cost, resulting in a net marginal milk value. Value of feed associated with marginal milk loss was computed using NASEM (2021) guidelines assuming NE_L_ density of 1.72 Mcal/kg of diet DM. At this energy density, DMI per kilogram of marginal milk was calculated to be 0.43 kg of DM of a diet assumed to cost $0.31/kg of DM. Total cost of feed required to support marginal milk reductions due to GN CM was subtracted from net value of marginal milk priced at $20/45.4 kg to estimate the value of marginal milk losses. Death-related losses were based on death rates of 7.4%, 3.0%, and 0.7% for CON, SP2, and SP5, respectively, which were values computed from study data during the first 90 d after enrollment. Mastitis-related culling losses were computed based on study data (25.2% for CON, 11.3% for SP2, and 18.4% for SP5 within 90 d from enrollment). Full value of replacement cows was assumed to be $2,200/cow. Using a spreadsheet-based calculator (Excel, Microsoft Corp., Redmond, WA), residual value of each cow that died was depreciated based on her parity, future predicted production, future predicted feed costs, and the acquisition cost of a new replacement animal (Overton and Eicker, 2022). Net market value of each cull cow was computed based on her value sold to beef and her parity group (Nguyen et al., 2022). By this method, older cull cows had less potential residual value, so it was less costly to replace them.

Derivative economic calculations based on experimental results are summarized in Table 1. Sources of cost (treatment cost, milk discard cost, marginal milk expense after removal of potential feed cost, death loss expenses, and net culling loss expenses) were totaled to estimate total GN CM-related costs. Recognizing that data from Bruno et al. (2024) showed greater benefit of treating moderate than mild CM caused by GN bacteria, it was assumed that implementing different treatment protocols in commercial dairies for cows where CM severity differences may be subtle and thus impractical. Therefore, calculations assessed the economic impact across levels of nonsevere GN CM.

**Table 1:** Partial budget evaluation of economics related to treating mild to moderate gram-negative mastitis

Item	Treatment group^1^		
	CON	SP2	SP5
DIM at enrollment, d ± SD	126.4 ± 84.1	103.6 ± 76.3	124.9 ± 83.5
No. of ceftiofur HCl tubes/cow	0	2	5
Cost/tube (www.valleyvet.com), $	—	5.47	5.47
Treatment cost, $/cow	0.00	10.94	27.35
Mean days milk withheld due to mastitis or treatment	6.9	7.1	9.4
Test day milk yield at treatment, kg/cow per d	41.1	41.1	41.1
Milk discarded, kg/cow	283.6	291.8	386.3
Milk value, $/45.4 kg	20.00	20.00	20.00
Milk discard cost, $/cow	125	129	170
Average milk yield, kg/cow per d	39.3	39.3	39.3
Milk loss due to gram-negative mastitis at 5% reduction after milk withhold, kg/cow per 90 d	163	163	158
Gross milk value at $20/45.4 kg	72	72	70
Feed cost, $/kg DM	0.31	0.31	0.31
kg feed DM required to produce 1 kg milk at 1.72 Mcal NE_L_/kg DM	0.43	0.43	0.43
Estimated feed cost to produce marginal milk, $/cow	22	22	21
Value of marginal milk after feed costs, $/cow	50	50	49
Death loss within 90 d, % of enrolled	7.4	3.0	0.7
Cow value, $/cow	2,021	1,969	1,757
Death loss expense, $/cow	150	59	12
Mastitis-related culling loss within 90 d, % of enrolled	25.2	11.3	18.4
Culling loss (depreciated value minus net salvage value)	894	832	894
Net culling loss expense, $/cow	225	94	165
Total mastitis-related expenses over 90 d from enrollment, $/cow	550	343	423
Difference from CON, $/cow	—	−207	−127

1 CON = nontreated negative controls; SP2 = ceftiofur HCl once daily for 2 d; SP5 = ceftiofur HCl once daily for 5 d.

In our study, average DIM when cows were diagnosed with GN CM was 118 d (median 108 d), or roughly within the first third of lactation (Table 1). Cost of mastitis therapeutic IMM treatments were $0.00, $10.94, and $27.35 for CON (0 tubes), SP2 (2 tubes), and SP5 (5 tubes), respectively. At a milk value of $20/45.4 kg, the cumulative value of milk withheld was estimated to be $125, $129, and $170, respectively, for CON, SP2, and SP5. Milk production reductions due to GN CM over 90 d (excluding the milk withholding period following treatment) were estimated to be 166 kg/cow for CON, 165 kg/cow for SP2, and 159 kg/cow for SP5. At $20/45.4 kg, gross milk value losses over 90 d were largely unaffected by treatments. As a result, feed cost to produce marginal milk was estimated to be between $21 to $22 for all treatments. Value of marginal milk after feed costs were removed was therefore estimated to be $50 for CON, $50 for SP2, and $49 for SP5.

Details about culled and dead animals within 90 d after enrollment are detailed in Table 2. Death losses were estimated to be $150, $59, and $12, respectively, for CON, SP2, and SP5 and were based on the total expected economic loss due to death based upon the estimated depreciated cow value at the time of loss. Total mastitis-related culling losses were estimated to be $225, $94, and $165 for CON, SP2, and SP5, respectively. Sources of loss were summed for each treatment, totaling $550 for CON, $343 for SP2, and $423 for SP5. Thus, over the first 90 d after study enrollment and compared with CON, the choice to treat nonsevere GN CM with ceftiofur HCl reduced costs among SP2 cows by $207 and among SP5 cows by $127. These calculations project the estimated return on investment from treating GN CM were 18.9:1 for SP2 and 4.6:1 for SP5.

**Table 2:** Enrollments and mastitis-related culled and dead animals within 90 d after enrollment

Item	CON^1^		SP2^1^		SP5^1^	
	Younger^2^	Older^2^	Younger^2^	Older^2^	Younger^2^	Older^2^
Cows enrolled, n (% of treatment total)	63 (46.7)	72 (53.3)	64 (48.1)	69 (51.9)	68 (46.3)	79 (53.7)
Total culled for all reasons ≤90 d, n	17	20	5	15	13	19
Total culled due to mastitis ≤90 d, n	14	20	4	11	12	15
Total dead ≤90 d, n	7	3	2	2	0	1

1 CON = nontreated negative controls; SP2 = ceftiofur HCl once daily for 2 d; SP5 = ceftiofur HCl once daily for 5 d.

2 Younger (lactation ≤2); older (lactation ≥3).

Treatment of CM caused by GN bacteria has been a subject of discussion, especially concerning the need to treat nonsevere cases with antimicrobials. One consideration when assessing whether to treat GN CM is medicine cost, which varies depending on the selected product and treatment duration (Pinzón-Sánchez et al., 2011). On its surface, the decision to not treat nonsevere GN CM is economically appealing because there is no medicine cost and, therefore, no mandated milk withdrawal time. However, other aspects must be considered in the decision-making process, and this subject was discussed in our previous article. Bruno et al. (2024) found that the average days to be considered clinically normal for CON cows was 5.6 d, which was not different from SP2 cows (5.5 d). Although all cows stayed in the hospital an extra day waiting for the results of OFC, cows in both treated groups (SP2 and SP5) required 3 d for the drug withdrawal. While there was no medicine cost for CON cows, there was no difference in milk withholding days for CON (6.9) and SP2 (7.1). Bruno et al. (2024) also found that rate of clinical cure (i.e., cows producing milk of normal appearance and absence of clinical signs following detection of mastitis and enrollment) in the CON group was lower (48.5%) than either SP2 (88.4%) or SP5 (89.4%), thus offering no cost advantage for the CON group.

Dairy producers tend to focus on managing direct costs when evaluating the cost efficiency of treating CM cases, but the economic impact due to indirect expenses associated with nontreatment of GN CM can be insidious. The USDA (2016) reported that mastitis is the reason for permanent removal of 16.5% of cows from large US dairy operations. Bruno et al. (2024) showed untreated cows were at higher risk for herd removal, which could result in higher mastitis-related costs for dairies. The proportion of cows that exited our study (mastitis-related culls and deaths) was higher for CON compared with SP2 and SP5 and higher than previously reported (Lago et al., 2011; Schukken et al., 2011; Vasquez et al., 2016; Fuenzalida and Ruegg, 2019), perhaps due to differences in dairy management decisions, replacement heifer availability, or economic conditions during the study. As with the aforementioned studies, we did not interfere or attempt to influence culling decisions, but merely reported the results.

Average DIM at enrollment into our study was within the first third of lactation, and 47% of cases occurred in young cows (first and second lactation). As pointed out by Bar et al. (2008), the cost of a case of mastitis is in part influenced by stage of lactation when diagnosed, with expenses in cows experiencing mastitis in early lactation being greater than for cows in later lactation. Economic inputs such as milk price, replacement cost, and treatment cost can vary from farm to farm over time. Cha et al. (2011) estimated the average cost per case of GN CM was $211.03, primarily due to milk loss ($152.76), treatment cost ($32.74), and decreased fertility ($25.54). Rollin et al. (2015) estimated the cost of a case of mastitis occurring in the first 30 d of lactation to be $444, which was attributed to direct expenses of $128 (diagnostics, therapeutics, nonsalable milk, veterinary expenses, labor, and death losses) and indirect expenses of $316 (milk yield loss, culling/replacement costs, future reproduction loss). Leite de Campos et al. (2023) performed a partial budget calculation and found that the average cost of CM (drugs + milk discard, including nontreated cases) was $192.36. Mastitis, in general, and GN CM, in particular, negatively affect reproductive function in dairy cows (Hertl et al., 2010). However, the impact of GN CM on reproduction was not considered in our computations because the observation interval relative to the occurrence of GN CM diagnosis was 90 d, and therefore, reproduction data were incomplete.

The productive lifespan of an individual cow is determined by biologic (death, disease, or reproductive failure) and by elective (performance-related) issues. The appropriateness and timeliness of management's response to these issues influence a herd's profitability (Dallago et al., 2021). Mastitis is one of the main reasons for culling early in lactation (Langford and Stott, 2012). Cows departing from a dairy prematurely contribute to financial losses due to the explicit and implicit losses associated with replacing a previously healthy and productive cow with a younger animal. Rollin et al. (2015) reported that premature culling and replacement represent the greatest cost of all categories they examined when evaluating mastitis-related culling. Mastitis-related culling rates for CON, SP2, and SP5 groups over 90 d after enrollment were 25%, 11%, and 18%, respectively; at a replacement cost of $2,200/cow, the replacement GN CM-related cost for the CON group was higher than for the 2 groups treated with ceftiofur HCl.

Previous research has documented the impact of mastitis on milk production following infection and established that the impact of GN CM on milk yield and culling risk is more significant than the impact of gram-positive mastitis (Schukken et al., 2009; Hertl et al., 2011; Heikkilä et al., 2018). Cows that contract CM are typically higher-producing cows than those that do not develop CM (Wilson et al., 2004). Heikkilä et al. (2018) investigated pathogen-specific impacts of mastitis on milk production of dairy cows in cows infected with 6 common udder pathogens and found that infections with *E. coli* diagnosed before peak lactation caused losses of 10.6% of the 305-d milk yield (3.5 kg/d). Gröhn et al. (2004) examined milk yield reductions following pathogen-specific CM and demonstrated that GN infections (*E. coli* and *Klebsiella* spp.) had more profound and prolonged negative impacts on milk yield than other organisms. Among primiparous cows with GN infections, milk production was suppressed by approximately 13% for 70 d following diagnosis. In comparison, among multiparous cows, reduction in milk yield per case of GN mastitis was 12.6% (Gröhn et al., 2004). Notably, these 2 studies included cows with all levels of mastitis severity, whereas our study included only cows with mild or moderate GN CM. Furthermore, milk production is an economically relevant outcome to consider when assessing the cost-effect relationships of mastitis treatments. Data from Bruno et al. (2024) did not offer an opportunity to assess the impact of GN CM on milk yield before, during, or after a GN CM diagnosis. However, previous studies have shown milk production decreases associated with GN mastitis but did not show persistent significant differences in milk production decreases or recovery times between treated and control cows (Schukken et al., 2011; Fuenzalida and Ruegg, 2019). Other factors associated with mastitis that contribute to the economic loss include additional labor demands, increased veterinary costs, and decreased milk quality, but we did not incorporate these factors into our study.

Our partial cost analysis calculations were based on outcomes from Bruno et al. (2024) and represent findings from cases observed in the 3 dairies in California. Culling is a complex process, and dairy farms consider many factors when making decisions. Although culling and replacement can be multifactorial, all treatment groups were faced with the same culling strategy by the farms, regardless of treatment. Our data indicate an association between the treatment group and culling and death, with higher removal rates within 90 d of mastitis in the control group than in the treated groups. Culling rates in our study were higher than those found in previous studies, possibly due to individual farm culling criteria or differences in economic conditions and opportunities. Acknowledging that a considerable portion of the calculation is based on death and culling rates, we recognize that the total cost would be different if we used mortality and culling rates found in previous studies in our calculations, and therefore, only utilizing data from 3 dairies could be perceived as a limitation of the present report. One constraint of this study is that most cases were caused by *E. coli*, and therefore, further research on other GN bacteria, particularly *Klebsiella* spp., is needed.

Despite medicine cost to treat cows, and based on rates generated from a previous study, total mastitis-related expenses over 90 d following enrollment were less for treated than for nontreated GN CM cases and more advantageous for cases treated for 2 d compared with 5 d. The proportion of cows that experienced mastitis recurrence was higher for CON, which led to use of more postenrollment IMM antibiotics. There was no difference between CON and SP2 in the number of days for the quarter to be considered normal and days of milk withholding. The proportion of cows that were culled or died due to the GN CM was higher for CON, suggesting treatment of nonsevere GN CM increased the likelihood that a cow will recover and remain a productive member of the herd for a longer duration. Furthermore, a 2-d treatment protocol yields lower treatment costs and fewer days of milk withholding.
